# CAR T Cell Therapy in Primary Brain Tumors: Current Investigations and the Future

**DOI:** 10.3389/fimmu.2022.817296

**Published:** 2022-02-21

**Authors:** Ya-Jui Lin, Leila A. Mashouf, Michael Lim

**Affiliations:** ^1^ Department of Neurosurgery, Stanford University School of Medicine, Palo Alto, CA, United States; ^2^ Department of Neurosurgery, Chang Gung Memorial Hospital, Linkou Medical Center, Taoyuan, Taiwan; ^3^ Harvard Medical School, Boston, MA, United States

**Keywords:** glioma, focus ultrasound, CAR T cell, brain tumor, immunotherapy

## Abstract

Chimeric antigen receptor T cells (CAR T cells) are engineered cells expressing a chimeric antigen receptor (CAR) against a specific tumor antigen (TA) that allows for the identification and elimination of cancer cells. The remarkable clinical effect seen with CAR T cell therapies against hematological malignancies have attracted interest in developing such therapies for solid tumors, including brain tumors. Glioblastoma (GBM) is the most common primary brain tumor in adults and is associated with poor prognosis due to its highly aggressive nature. Pediatric brain cancers are similarly aggressive and thus are a major cause of pediatric cancer-related death. CAR T cell therapy represents a promising avenue for therapy against these malignancies. Several specific TAs, such as EGFR/EGFRvIII, IL13Rα2, B7-H3, and HER2, have been targeted in preclinical studies and clinical trials. Unfortunately, CAR T cells against brain tumors have showed limited efficacy due to TA heterogeneity, difficulty trafficking from blood to tumor sites, and the immunosuppressive tumor microenvironment. Here, we review current CAR T cell approaches in treating cancers, with particular focus on brain cancers. We also describe a novel technique of focused ultrasound controlling the activation of engineered CAR T cells to achieve the safer cell therapies. Finally, we summarize the development of combinational strategies to improve the efficacy and overcome historical limitations of CAR T cell therapy.

## Introduction

T cells engineered to express chimeric antigen receptors (CAR T cells) have shown remarkable efficacy in treatment of hematological cancer and represent a promising frontier for innovation in their application to treat solid malignancies (1). Chimeric antigen receptors (CARs), which are assembled by the fusion of a recognition domain, single-chain antibody and T cell stimulatory domain, can be engineered to recognize a target antigen without major histocompatibility complex (MHC) presentation (2, 3). These CAR constructs are then transfected into T cells using plasmids, mRNA, or viral vector transduction to ultimately display on the cell surface. CAR T cells can be engineered to target virtually any antigen, such as tumor-associated antigens or microbial antigens. CAR T cells can become activated without the contribution of antigen presenting cells and MHC molecules, which greatly contributes to their effectiveness in activating the immune system. Other costimulatory receptors such as CD28 or OX40 may be added to further improve the T cell response. Once activated, CAR T cells can individually activate multiple immune cells and additionally secrete cytokines that promote cell trafficking and effector function, amplifying their individual effect.

The first CAR T cells were developed in 1987 by Kuwana et al. (4). Through iterations over the following decades, CAR T cells progressed through second, third, and fourth generation compositions which improved their antitumor activity, effector function and *in vivo* persistence, with expanded modifications allowing for enzymatic degradation of extracellular matrix for solid tumors and costimulation of various receptors with additional ligands (1–3). First generation CARs consist of a single-chain variable fragment antigen recognition domain, transmembrane component, and intracellular T-cell activation domain akin to the CD3 zeta chain (2). Second generation CARs incorporate a costimulatory domain, such as CD28 or 4-1BB, and are utilized in current commercially available CAR T cell therapies. Third generation CARs combine two distinct costimulatory domains into their intracellular structure. Fourth-generation CAR T cells are also known as TRUCKs or “armored CARs” (CAR-redirected T cells that can serve as a delivery platform for transgenic products) (1, 3). These modifications incrementally improved CAR T viability as a therapy for cancers, with particular success in hematological cancer. CAR T therapies have been developed for chronic lymphoblastic leukemia (CLL) and acute lymphoblastic leukemia (ALL), as well as recurrent lymphoma and prostate cancer, and investigation continues for optimizations that prove clinical effectiveness in other malignancies (1).

## Car T Cell Therapy In Hematologic Cancer

CAR T cell therapy gained its initial foothold for hematogenous cancers, including CLL and ALL, and have rapidly changed the landscape of treatment for acute and chronic B cell leukemias with further indications in lymphoma and myeloma being explored (1, 3, 5). In August of 2017, the first CAR T cell therapy was approved by the Food and Drug Administration (FDA) for treatment of B-cell ALL (1). This therapy, called tisagenlecleucel-T targeting CD19, demonstrated efficacy in preliminary results from the Phase 2 multicenter ELIANA trial (1, 3). Impressively, this study demonstrated a 60% complete remission rate and 81% overall response rate in 75 children and young adults with durable response reported at 80% 6-month relapse-free survival associated with prolonged detection of CAR T cells in peripheral blood samples and persistent B-cell aplasia. Toxicity was common among study participants however, with 73% of patients experiencing severe adverse events (grade 3 and above) (3).

Within the year following the approval of tisagenlecleucel-T, two more therapies were approved by the FDA (2). Based on the Phase 2 multicenter ZUMA-1 trial, the first CAR T cell therapy, known as axicabtagene ciloleucel, was approved in October 2017. Results from this trial demonstrated 83% overall response rate and 58% complete remission rate among 101 participants, with 39% of patients with prolonged durable response at median follow-up of 27.1 months. Toxicity was less common in this trial than the ELIANA trial, with 48% of patients experiencing severe adverse events and 11% with cytokine release syndrome (CRS) compared to 47%, although a greater percentage of patients suffered neurotoxicity (NT) at 32% compared to 13.3% (3).

The success of the JULIET trial led to the approval of tisagenlecleucel for a second indication, relapsed and refractory lymphoma in 2018 (3). The JULIET trial showed 40% CR and 100% durable response at median follow-up of 29.3 months among a study population of 93 patients with diffuse large B-cell lymphoma (DLBCL). Toxicity overall was similar to the ELIANA trial, specifically measuring NT at 12% and CRS at 22% (3).

In another investigation targeting DLBCL, the Phase 2 TRANSCEND trial, lisocabtagene maraleucel was shown to be effective with 80% ORR and 55% CR with a 6-month durable remission of 50% (2). Toxicity in this study was remarkably low with only 1 patient experiencing CRS and 12% of patients experiencing neurotoxicity (3).

The success of CAR T cell therapy against hematogenous cancer is clear and represents tremendous progress in the treatment of these malignancies. Future progress in decreasing the toxicity profile associated with CAR T cell therapy, along with improvements in response rate, durability of remission, and CAR T exhaustion, will be catalytic to more widespread use to greatly improve patient prognosis (1–3, 6).

## Car T Cell Therapy In Solid Tumors

Inspired by the initial success against hematological cancer, trials of CAR T cells therapy for solid tumors were instigated, though yielded less impressive results (5). The growing number of clinical trials focused on solid tumors include CAR T cells targeting carcinoembryonic antigen (CEA), the diganglioside 2, mesothelin, interleukin 13 receptor alpha (IL-13Ralpha), human epidermal growth factor receptor 2 (HER2), fibroblast activation protein (FAP), and L1 cell adhesion molecule (L1CAM). Although the number of trials here is impressive, the most successful of these trials report complete remission of 3 of 11 patients using GD2 CARs for neuroblastoma, stable disease in 4 of 17 patients using HER2 CARs for sarcoma, and partial response in 2 of 11 patients in HER1 CARs for lung cancer. The differential success of CAR T therapy in solid tumors thus far compared to hematologic cancer has prompted investigation into possible explanations and identification of unique limitations that may not exist for treatment of hematologic cancers (5).

Proposed challenges in the treatment of solid tumors include identifying highly and uniformly expressed tumor antigens (TAs), CAR T cell trafficking from blood to solid tumor sites, stromal infiltration, TA loss, inherent tumor heterogeneity, and the immunosuppressive tumor microenvironment (TME) (5). Current strategies to optimize CAR T-therapy in solid tumors generally fall within categories of disrupting immunosuppressive axes, autocrine stimulation, remodeling and induction of endogenous immune responses, and enhanced tumor infiltration. Development of armored CARs, or fourth generations CAR T cells, with unique immunostimulatory mechanisms specifically targets the hurdle of the TME. Some examples of fourth generation CAR T cells include candidates that express CD40 ligand, secrete IL-18, and secrete a PD-1-blocking single chain variable fragment (scFvs) (3, 5).

Trafficking of CAR T cells to the tumor site depends on the appropriate matched expression of adhesion molecules and chemokine receptors (such as CXCR3 and CCR5) that allow for endothelial adhesion and transport, along with tumor-specific targeting (3, 5). Appropriate design of CAR T cells to achieve these requirements, particularly as antigen expression greatly varies from tumor-to-tumor, has been notably challenging. Additional physical and anatomical barriers represented by the tumor stroma and high intratumoral pressure, along with unique physical barriers such as the Blood-Brain Barrier (BBB) to treating intracranial malignancies, necessitate further innovation to improve viability of CAR T cell therapy for solid tumors (5). Current investigations to improve trafficking to the tumor site include use of oncolytic viruses armed with chemotactic chemokines to attract CAR T cells and local administration (5, 6). Strategies to adequately disrupt physical barriers include targeted digestion of the dense tumor extra-cellular matrix, which demonstrated success in xenograft models using CAR T cells expressing heparinase, and ultrasonic disruption, such as focused ultrasound (FUS) applied to disrupt the BBB to deliver therapeutics to intracranial malignancies (3, 5, 6).

## Car T Therapy For Brain Tumors

Brain tumors, including primary and metastatic neoplasms, have a great impact on neurological function and quality of life, particularly in cases of more aggressive or malignant neoplasms (7). Advances in imaging instruments, such as computed tomography (CT) and magnetic resonance imaging (MRI), have led to an increase in incidence of brain tumors being diagnosed (8). Within gliomas, glioblastoma (GBM) is most aggressive and malignant with a median overall survival of 14 to 17 months despite standard of care (surgery, radiotherapy, and chemotherapy) (9, 10). Tumor-treating fields, a new FDA-approved therapeutic strategy, has shown promise in extending the overall survival to 20.9 months (11).

Although previously believed to be immune-privileged, the brain is now known to be immunologically dynamic, though quiescent at baseline. The BBB and resident microglia are the first lines of defense in CNS. Immune cells, such as dendritic cells (DCs), lymphocytes, and monocytes, are mostly absent in the CNS during the quiescent period (12). Additionally, the CNS was thought to be lacking conventional lymphatics (13) until 2015 when lymphatic-like structures were discovered along the dural venous sinuses in rodents (14). Both local and systemic immune cells can detect antigens from the CNS; peripheral immune cells are then able to cross the BBB after detection of these danger signals to induce further inflammatory responses, providing also a significant mechanism for targeted immunotherapy against brain tumors (15).

**Figure 1:**
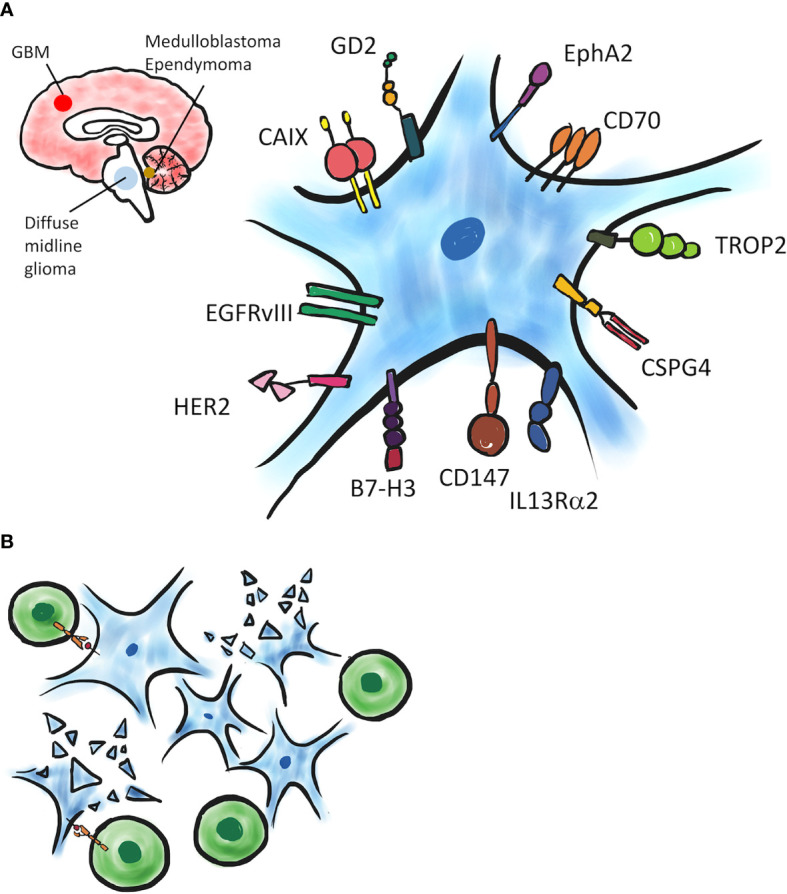
A schematic of CAR T targeting brain tumors. **(A)** Several TAs expressed on tumor cells have been evaluated in preclinical and clinical CAR T studies. **(B)** TA-expressing tumor cells can be recognized and eliminated by TA-specific CAR T cells.

Advances in immunotherapy have increased the therapeutic options for patients with brain cancers. There are 70 clinical trials testing immunotherapy for either primary or secondary brain tumors currently. Although immunotherapy for GBMs is not included in standard of care based on European Association of Neuro-Oncology (EANO) guidelines, ICIs-based immunotherapy is recommended as tumor-specific adjuvant therapy for brain metastases (16). CAR T therapy is a promising strategy to treat hematological malignancies and some kinds of solid tumors. CAR T cells can specifically recognize cancer cells, due to their functionalization with homing surface molecules, and exert targeted cytotoxicity. An ideal TA target should be expressed homogenously on all cancer cells within a primary tumor and metastases. Additionally, in order to avoid killing of normal cells by CAR T cells, the TA should be undetectable or minimally expressed on normal tissues. Non-specific targeting of normal cells leads to toxicity and CRS, which is potential significant side effect of CAR T cell therapy. CAR T cell therapy utilizing specific TAs has been explored to treat GBMs and pediatric brain tumors. In this review, we will examine current clinical and preclinical study of CAR T cell against GBMs and pediatric brain tumors (**Figures 1A, B**).

### Glioblastoma

Several molecules have been identified as potential TAs for CAR T cell therapy targeting GBMs through immunohistochemical (IHC) analysis. Here, we will discuss current CARs which have been used in clinical trials and preclinical models of GBM

#### Completed Clinical Trial of Targets

##### EGFRvIII

EGRFvIII, a mutated Epidermal Growth Factor Receptor (EGFR) from an in-frame deletion of exons 2 to 7, is the most common variant of this receptor in cancers (17, 18). In GBMs, approximately 40% of newly diagnosed patients have amplification of the EGFR gene, and about 50% of EGFR-amplified GBM patients exhibit constitutively oncogenic EGFRvIII (19, 20). The structure of the extracellular domain altered by this mutation can be targeted as a unique epitope by specific monoclonal antibodies (mAbs) with limited likelihood of on-target/off-tumor toxicity (21). Therefore, both vaccine and CAR T cell therapies targeted to EGFRvIII have been developed (22, 23). In preclinical studies, EGFRvIII CAR T cells demonstrated excellent reduction of tumor growth (24). However, limited efficacy has been shown for CAR T cells specific to EGFRvIII in GBM patients (25). To determine the extent of EGFRvIII CAR T cell trafficking to the tumor, surgical specimens were evaluated in one study for EGFRvIII expression across the TME after EGFRvIII CAR T cell administration. This study found EGFRvIII loss or decreased expression in resected tumors of most patients treated with CAR T cell infusion (23). These data demonstrated that the heterogeneous expression of EGFRvIII in glioma cells limited the efficacy of EGFRvIII CAR T cell therapy, and further results in generation of escape variants resistant to the same therapy (21). Regarding the TME, in post-CAR T cell surgical specimens, the phenotypic analyses of non-transduced and polyclonal T cells showed significant infiltration of regulatory T cells (Tregs). Additionally, excessive upregulation of immune checkpoints and immunosuppressive molecules, such as programmed death (PD) ligand 1 (PD-L1), indoleamine 2,3-dioxygenase-1 (IDO-1), transforming growth factor–β (TGF–β), and IL-10, was demonstrated by IHC (23). This suggests that EGFRvIII CAR T cells induced a compensatory immunosuppressive response in the TME, implying EGFRvIII CAR T is possibly more effective when combined with other immunotherapy to enhance immunity or reprogram the TME.

##### IL13Rα2

The function of IL-13 is to regulate inflammation and the immune response with binding to IL13Rα1. Additionally, IL-13 binds to the high affinity decoy receptor IL13Rα2 (26). IL13Rα2 is expressed in over 75% of GBMs and is related to tumor aggressiveness and poor prognosis. However, it is not expressed significantly in normal brain parenchyma or most normal tissues, except the testes (27). Due to the specificity of IL13Rα2 for GBM, IL13Rα2 has long been used as a potential candidate as a target for CAR T cell therapy (26). In the first-in-human pilot study, twelve intracranial infusions (maximum dose 1 x 10^8^) of IL13Rα2 CAR T cells were administrated into three patients with recurrent GBM, showing fair tolerance with delivery of CAR T cells and excellent antitumor response in two of the three treated patients. Some mild adverse events such as headaches and transient neurologic deficits occurred but subsided after management (28). Another trial of IL13Rα2 CAR T cells incorporated positron emission tomography (PET) imaging to check the trafficking of CAR T cells into brain parenchyma (29). Because the uptake of [18F]FHBG (9-[4-[18F] fluoro-3 (hydroxymethyl)butyl]guanine) in HSV1-tk expressing cytotoxic T lymphocytes is higher than in naïve T cells, it can be used as a tracer for IL13Rα2 CAR T cells expressing an HSV1-tk reporter gene. This study demonstrated the approach is safe and feasible with an obvious increase of [18F]FHBG activity demonstrating T-cell trafficking to tumor regions.

##### HER2

Human epidermal growth factor receptor 2 (HER2), a receptor tyrosine kinase, was found overexpression in many kinds of cancers and approximately 80% of GBMs (30, 31). A recent paper demonstrated that third generation HER2 CAR T cells can target and kill GBM cells *in vitro*. Additionally, significant improvement of efficacy was found when combined with PD-1 blockade (32). However, safety concerns were raised due to the death of a colorectal cancer patient who received 1 x 10^10^ third-generation HER2 CAR T cells (with a trastuzumab-based antigen-recognition domain and a CD28.4-1BB signaling domain) (33). Following these concerns, up to 1 x 10^8^ second-generation HER2 CAR T cells (an FRP5-based exodomain and a CD28 signaling endodomain) was administrated in GBM patients with no dose-limiting toxic effects observed (34). After infusion, HER2 CAR T cells could be detected by qPCR in all patients with peak timing of 3 hours in 15 of 17 patients. In the remaining 2 patients, peak timing was found to be one week and two weeks respectively. Blood levels declined every month and 2 cases remained positive at 12 months. However, none were positive at 18 months. These findings demonstrated HER2 CAR T cells did not expand after administration but persisted for 1 year. An important point of this study was to utilize CAR-modified T cells which were specific for adenovirus, cytomegalovirus (CMV), or Epstein–Barr virus (EBV). The CAR T cells were generated from virus-specific T cells and potentially provided a co-stimulatory effect under latent virus antigen recognition (34, 35). The major concern of HER2 CAR T cell is risk of on-target off-tumor toxicity because of HER2 expression in some normal tissues of vital organs (33). However, a phase I clinical trial has shown that virus-specific CAR T cells through peripheral infusion in GBM patients are safe and feasible (34), and demonstrated promising results with efficacy.

#### Ongoing Clinical and Preclinical Studies of CAR T Cell Therapy

CAR T cells targeting several TAs (ex. B7-H3, CD147, GD2) are under investigation in clinical trials currently (**Table 1**). B7-Homolog 3 (B7-H3, as known as CD 276) and PD-L1 (B7-H1) are among the B7 family of immune checkpoint molecules (36). B7-H3 is not only highly expressed on tumor cells in most types of solid cancer (37), but also expressed in vessels and fibroblasts within tumors. This implies that B7-H3 CAR T cells can eliminate tumor cells through direct targeting, disrupt stroma, and inhibit neo-angiogenesis, as well (38, 39). CD147, a 57-kilodalton (kDa) type-I transmembrane protein, is one of the immunoglobulin superfamily of adhesion molecules. It induces metalloproteinases-1, -2, -3, -9, -14, and -15 released by fibroblasts, and further results in extracellular matrix (ECM) degradation, tumor progression, invasion and metastasis (40). CD147 is highly expressed in GBM, associated with poor prognosis in patients, compared to normal brain (41, 42). Disialoganglioside (GD2) is also highly expressed on several cancers including neuroblastoma, retinoblastoma, and melanoma (43). GD2 is an attractive target TA for GBM therapy with high expression demonstrated on GBM cell lines and patient samples (44). There are several ongoing clinical trials of CAR T cell therapy to B7-H3, CD147, and GD2. For B7-H3, a randomized, parallel-arm, phase I/II study (NCT04077866) is in progress to evaluate the safety and efficacy in patients of refractory or recurrent GBM between cycles of temozolomide (TMZ). For CD147, a single-center, single-arm, open label and dose escalation clinical study (NCT04045847) in patients with recurrent GBM is in progress. For GD2, a phase I clinical study (NCT04099797) of CAR T expressing GD2 for treatment of patients with GD2 expressing brain tumors, such as high-grade glioma (HGG) including GBM, diffuse intrinsic pontine glioma (DIPG), medulloblastoma, or other rare brain cancers, is underway.

**Table 1:** Ongoing clinical trials of CAR-T therapy for glioma.

Molecular target	Clinical trial title	Study phase	CAR-T cell dosage and combination regimen	Sponsor/site
**B7-H3**	B7-H3 CAR-T for Recurrent or Refractory Glioblastoma	I/II	CAR T cells delivered intratumorally or intracerebroventricularly for three doses between temozolomide cycles	Second Affiliated Hospital of Zhejiang Ningbo
				Yinzhou People’s Hospital, Huizhou Municipal Central Hospital, BoYuan
				RunSheng Pharma (Hangzhou) Co., Ltd. (China)]
**CD147**	CD147-CART Cells in Patients With Recurrent Malignant Glioma	I	CAR T cells injected into tumor cavity once a week for three weeks	Xijing Hospital
**GD2**	C7R-GD2.CART Cells for Patients with GD2-expressing Brain Tumors (GAIL-B)	I	CAR T cells (1x10^7^ -1x10^8^) delivered *via* intravenous administration with or without lymphodepletion chemotherapy	Baylor College of Medicine (Center for Cell and Gene Therapy, Baylor College of Medicine)

Recently, several molecules have been investigated as CAR Targeting T cell therapy in preclinical glioma models (**Table 2**). The results are promising, however, the safety concerns and on-target off-tumor toxicity still require further preclinical investigation and clinical trials to determine their severity.

**Table 2:** Preclinical study of CAR-T therapy for glioma.

Molecular target	Characteristics	Studies
**CAIX** (Carbonic anhydrases IX)	induced under hypoxic conditions overexpressed in solid tumors including GBMs (45)	CAR T cells induced cytotoxicity in GBM cells with survival benefit in mice (46)
**CD70**	type II transmembrane protein binding to CD27 expressed on activated T cells and mature DCs expressed on certain solid tumors including GBMs constitutive CD70 expression on GBM cells cause an immune escape by promotion of T cell death (47)	CAR T cells target and lead CD70^+^ GBM cells to death *in vitro*, and no toxicity in xenograft and syngeneic models (48) effective in glioma and head and neck cancer by CD70-specific CAR T cells (48)
**CSPG4** (Chondroitin Sulfate Proteoglycan 4)	related with cell proliferation/migration *in vitro*, and metastatic spread *in vivo* expression level correlated inversely with survival period in glioma patients (49)	highly expressed in GBM tissue and tumor associated vessels, without detection in healthy brain tissues (50) CAR T cells with intracranial delivery could inhibit tumor progression in orthotopic GBM neurosphere xenograft models (50)
**EphA2** (Erythropoietin-producing hepatocellular carcinoma A2)	Eph family of receptor tyrosine kinases (RTKs) correlated with tumorigenesis, invasion, angiogenesis and metastasis (44, 51)	kill differentiated GBM cells and GBM cancer stem-like cells *in vitro* with survival benefit in orthotopic xenograft SCID mouse models (52) Great anti-glioma activity (53)
**TROP2** (Trophoblast cell surface antigen 2)	36 kDa transmembrane glycoprotein highly expression over certain solid tumors (54, 55), a stem cell marker (56) high TROP2 expression on GBM cells, however, low expression on normal brain parechyma (57)	highly expressed in breast, pancreas and prostate cancer cells (58) the recognition and elimination of GBM cells by CAR T cells is under investigation.

### Pediatric Brain Tumor

Pediatric brain cancers remain among the leading causes of cancer-related death in children and thus necessitate urgent development of new therapies (51). CAR T cell therapy represents a promising approach in pediatric brain tumors as they can theoretically be specifically directed to tumor cells with limited cytotoxicity in normal tissues.

#### Medulloblastoma

Medulloblastoma is malignant brain tumor with highest prevalence in the pediatric population. The incidence rate is approximate 6.0 per million in 1 to 9-year-old patients (59). This tumor develops in the cerebellar vermis and thus are common posterior fossa tumors (60). Based on 2021 WHO classification, medulloblastoma is categorized into four groups based on its distinct molecular subtypes, including WNT-activated, SHH-activated and TP53-wildtype, SHH-activated and TP53-mutant, and non-WNT/non-SHH (61). The classification also directs potential therapeutic targets. The prognosis of medulloblastoma is related with histology and molecular diagnosis, metastatic status, and age (61, 62). Thus far, the current standard of care consists of surgery, chemotherapy, and craniospinal irradiation. CAR T cell therapy provides an alternative strategy for treating medulloblastoma, with several target antigens currently under investigation.

HER2 expression can be estimated approximately 40% of medulloblastomas (63) but there is no expression in normal brain tissues (64). Therefore, it is likely an ideal target antigen for CAR T cell therapy for medulloblastoma. First generation CAR T cells targeting HER2 (without co-stimulatory domain) demonstrated good tumor targeting and tumor regression in orthotopic xenogeneic medulloblastoma mice model (65). Second generation CAR T cells (with 4-1BB co-stimulatory domain) exhibit increased T cell activation and down-regulate T cell exhaustion with improved persistence of CAR T cells (66, 67). B7-H3 CAR T cells also demonstrated benefit in xenograft models of medulloblastoma, pediatric osteosarcoma, and Ewing sarcoma (68). Due to heterogeneity of antigen expression, multivalent CAR T cell targeting EPHA2, HER2, and IL13Rα2 were created, showing positive results in preclinical models of recurrent medulloblastoma and GBM (69, 70).

#### Pediatric Ependymoma

Ependymomas are the third most common (about 5.2%) in pediatric brain tumors (30). Ependymomas are classified into molecular groups based on pathological histology, molecular features, and anatomic site (supratentorial, posterior fossa, and spinal compartments) (61). Posterior fossa ependymomas are categorized into 2 different groups, group A (PFA) and group B (PFB), each having distinct characteristics, epigenetics, and prognosis. PFA tumors are typically only diagnosed in infants, while PFB tumors are found equally in adults and adolescents (71). PFA patients suffer from higher recurrence after treatment and worse overall survival (72). Standard of care includes surgical resection and radiotherapy. Aggressive gross total resection is important to prevent recurrence, but it is sometimes difficult due to local infiltration. The 5-year overall survival is as low as 37% for recurrent tumors (73). It is clear that new strategies, including immunotherapy, are needed for treatment of ependymoma. EphA2, IL13Rα2, HER2, and Survivin molecules are expressed specifically in ependymomas (74, 75), and have been shown potentially target for CAR T cell therapy. Trivalent CAR T cells targeting to HER2, IL13Rα2, and EphA2 demonstrated efficacy in xenograft ependymoma models (69).

#### Pediatric High-Grade Gliomas

Pediatric High-Grade Gliomas (pHGGs) make up less than 20% of pediatric brain tumors. Based on 2021 WHO classification, they are classified to four types, including Diffuse midline glioma (DMG) H3 K27-altered, Diffuse hemispheric glioma H3 G34-mutant, Diffuse pediatric-type high-grade glioma H3-wildtype and IDH-wildtype, and infant-type hemispheric glioma (61). DMG H3 K27-altered arise in midline regions such as thalamus, brainstem, and spinal cord, lending to their inoperability. Thus far, no standard therapy for DMG has been proven to be beneficial, though radiotherapy, targeted chemotherapy, and several strategies with mechanism of cell cycle inhibitor or anti-angiogenesis are treatment options (76–80). CAR T cell therapy for pHGGs is emerging as a result of translational research from adult GBM. In preclinical models of H3K27M-mutated DMG, GD2 CAR T cells intravenous (IV) administration could cleared most of engrafted tumors (81). Autologous GD2 CAR T cells for H3K27M^+^ DMG is ongoing as a phase I clinical trial (NCT04196413). GD2 CAR T cells at dose level 1 (1 million cells/kg IV) demonstrated not only improved or subsided neurological deficits and improved radiographic images, but also no evidence of on-target off-tumor toxicity. In this clinical trial, CAR T cells could be detected in CSF and blood, demonstrating successful trafficking to the CNS (82). In addition, B7-H3 also serves as a target for CAR T cells in clinical trials (NCT04185038, NCT0409979) due to high expression in DMG. CAR T cells targeting GD2 investigated in brain tumors, including neuroblastoma, proved to be well tolerated (83). B7-H3 CAR T cells significantly improved survival in preclinical medulloblastoma and DIPG xenograft mice models (68). Due to the heterogeneity of glioma, multivalent CAR T cell therapy designed to prevent antigen escape in pHGGs is of considerable benefit (84).

## Focused Ultrasound to Mediate CAR T Function

The most common complication of CAR T cell therapy is CRS, which usually occurs 1-2 weeks after initial administration (85, 86). Large-scale activation of CAR T cells leads to excessive inflammatory cytokines release, subsequently resulting in hypotension, fever, tachycardia, and even death from multiple organ failure (87). Administration of CAR T cell into the brain also introduces potential risk of neurotoxicity. Therefore, in addition to selection of optimal antigens for specific CAR T binding, mediating the functions of CAR T cells is another potential way to decrease toxicity of CAR T cell therapy.

Recently, focused ultrasound (FUS) has proven to be an innovative tool widely applied clinically to tumor thermo-ablation, brain-blood barrier opening with microbubbles for enhanced drug delivery, neuromodulation, and transgene expression (88–92). Notably, a FUS-based approach utilized acoustogenetics technology to activate CAR T cells with high precision control at the confined location of solid tumors (92) by transducing ultrasound signals into cellular activations and even genetic activation for therapeutic practices. This technique can decrease on-target off-tumor toxicity of CAR T cell therapy. Through the heat generated by short pulses of FUS, heat-induced reporter genes can be activated with high efficiency. In this study, the Cre-mediated gene was employed as a switch to deliver outputs of genetic activities from FUS inputs. Based on this mechanism, heat-inducible CAR expression and further functional reaction were proved *in vitro* Jurkat and primary T cells. Furthermore, MRI-guided FUS was utilized to induce gene activation *in vivo*. The FUS-inducible cytotoxicity of engineered CAR T cell has been shown significant tumor regression but significant less cytotoxicity in non-FUS-treated sites. Once CAR T cells leave the tumor site without further FUS stimulation, they will lose their CAR expression gradually. This leads to less on-target off-tumor toxicity of FUS-CAR-T-cells than with standard CAR T cells. Thus, this modular acoustogenetic approach with switchable target CAR genes can aimed at different cancers. Acoustogenetics, with advantages of direct and non-invasive control, may provide a broadly applicable method for genetically engineered cell therapeutics.

## Strategies to Enhance CAR T Cells Therapy

Although CAR T cell therapy has showed remarkable clinical response toward CD19 hematological malignancies, the benefit for solid tumors has been modest due to several challenges, such as insufficient trafficking to the tumors, defective recognition of the targeted TA, on-target off-tumor toxicity due to expression of the targeted TA in normal tissues, limited persistence and low proliferation in the TME, and the immunosuppressive TME. For CNS malignancies, penetration through BBB into tumor sites represents an additional hurdle. The BBB is a physiological barrier consisted of specialized endothelial cells joined by tight junction, with pericytes and astrocytes forming additional hurdles. Systemic administration of CAR T cells have shown limited accumulation in CNS tumors (79), necessitating further innovation in the delivery of CAR T cells to treat GBM. In several preclinical models of brain tumors, locoregional administration of CAR T cells increased T cell infiltration in tumor site with better tumor control (93–95). The findings support intratumoral or intracavitary injection of CAR T cells into the tumor or the resected cavity of tumor, or intracerebral/intraventricular injection into the brain parenchyma or cerebral ventricle, as viable strategies to mediate historical limitations.

**Figure 2:**
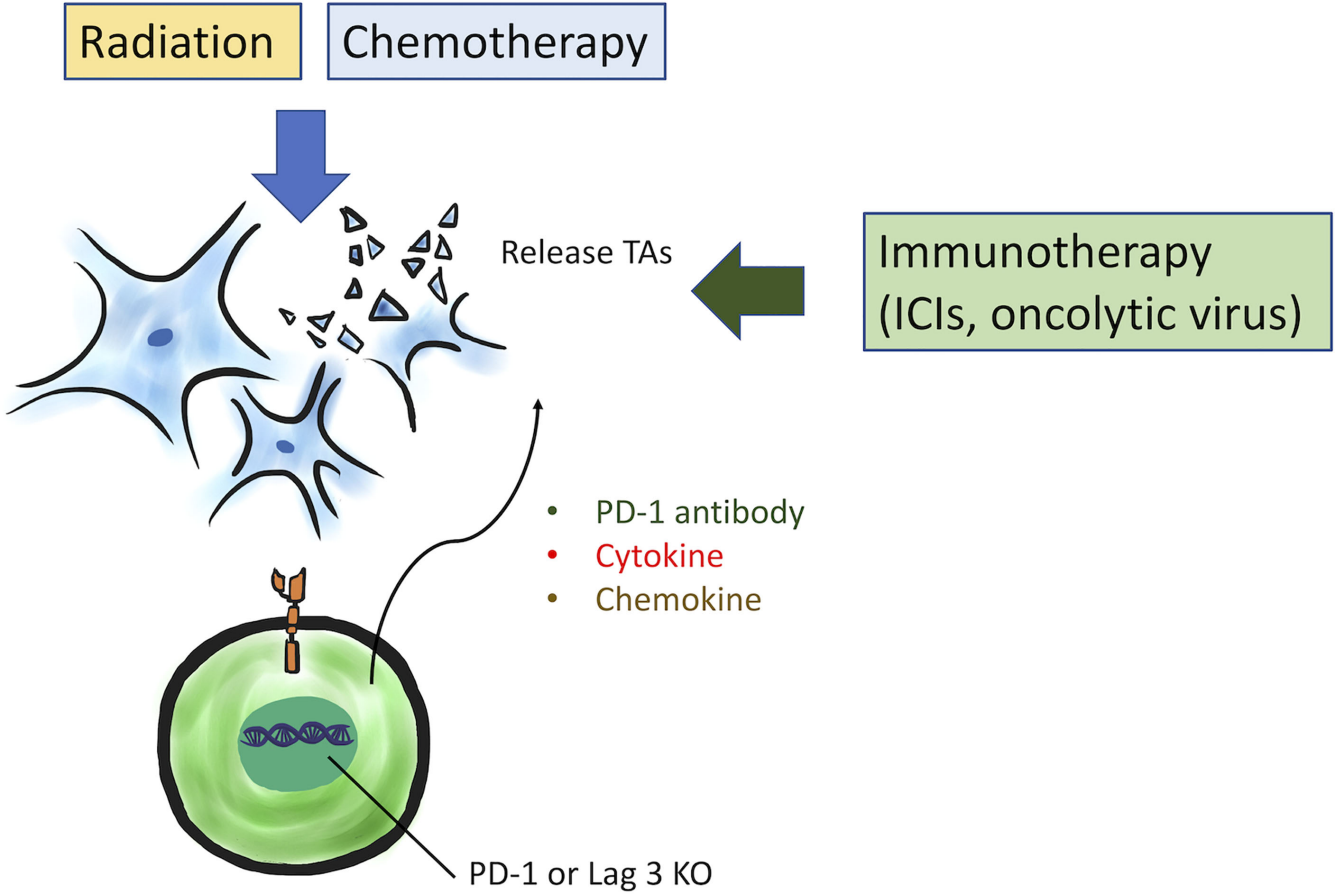
Several strategies to enhance CAR T cell functions are under investigation, including expression of cytokine and chemokine, induction of PD-1 antibody release, or PD-1 and Lag3 genes knockdown by CRISPR/Cas9 technology. Additionally, combination therapy with radiation or chemotherapy can cause tumor necrosis or apoptosis to increase TAs. Immunotherapy, such as ICIs and oncolytic viruses, could overcome the immunosuppressive TME to increase CAR T cell efficacy synergistically.

Alternatively, enhancing the function of CAR T cell constructs and targeting multiple TAs may also mitigate barriers to effective treatment (**Figure 2**). The strength and potency of CAR T antitumor activity has been already enhanced in previous studies *via* addition of costimulatory domains and functional moieties. Further engineering of the CAR to induce or secrete cytokines could additionally increase activity and persistence of CAR T cells (96). To enhance T cell trafficking into tumor site, engineered CAR T cells can also express chemokine receptors. For example, CD70-specific CAR T cells with CXCR1 and CXCR2 modification have demonstrated improvement of T cell trafficking and efficacy in tumor control in preclinical models of GBM (97). Additionally, disruption of immune checkpoint signaling within CAR T cells has been investigated. CAR T cells were engineered to release PD-L1 antibody (98) or to knock down PD-1 and Lag3 genes by CRISPR/Cas9 technology (99, 100). In addition, the hypoxia transcription factor HIF-1a subdomain can be incorporated in a CAR construct to reduce on-target off tumor toxicity, ensuring CAR T cells only activate under hypoxic conditions such as within the TME (101).

TA expression is frequently heterogeneous on many solid tumors, including GBMs, thus tumors may escape CAR T cell recognition and elimination due to antigen escape with increased risk of recurrence (79). In order to overcome antigen escape, CAR T cells targeting multiple TAs were developed (102). Trivalent CAR T cells targeting HER2, IL13Rα2, and EphA2 could cover more TAs and have showed significant survival benefit in mice bearing patient-derived GBM xenografts (70).

Combination therapy may also improve CAR T cell therapeutic viability in GBM (**Figure 2**). Radiotherapy and chemotherapy are usually standard therapeutic options for solid tumors and could be theoretically combined with CAR T cell therapy to target multiple mechanisms of oncogenesis. Radiotherapy can shape the TME to boost CAR T cell efficacy. First, radiation facilitates cytotoxic CD8^+^ T cells to recognize and eliminate cancer cells by increasing the expression of TAs (48). Then, tumor necrosis and apoptosis caused by radiation release more danger signals, and further increase infiltration of immune cells in TME *via* release of proinflammatory cytokines (IFNγ) or chemokine ligands (103, 104). Therefore, combination with radiotherapy demonstrated improved efficacy of CAR T cell therapy in some solid tumors including GBMs (48, 105, 106). Chemotherapy also has a similar ability to shape the TME to enhance CAR T cell efficacy by upregulation of TAs (107) and elimination of immunosuppressive cells (108). Exploration into suitable combinations of chemotherapy and CAR T cell therapy is underway.

Immunotherapy is another exciting candidate for combination therapy with CAR T cells, such as immune checkpoint inhibitors (ICI) or oncolytic viruses. Anti-PD-1/PD-L1 and anti-CTLA4 antibodies have demonstrated to increase CAR T cell efficacy in preclinical models (109). A clinical trial of IL13Rα2 CAR T cells with nivolumab (anti-PD-1) and ipilimumab (anti-CTLA4) showed synergic effect for recurrent GBMs (NCT04003649). Oncolytic viruses can cause immunogenic cell death with induction of a type I IFN response in the TME, and systemic innate and adaptive immune responses are activated consequently (110). The response in the TME to oncolytic viral therapy is promising as a concurrent enhancement of CAR T cell activity (111). For example, IL-7-loaded oncolytic adenovirus (oAD-IL7) combined with B7-H3 CAR T cells for the preclinical GBMs mice model is under investigation and has shown synergic survival benefit with tumor regression (112).

## Conclusion

CAR T cell therapy is a promising strategy for treatment of solid tumors. TA selection is important to target cancer cells specifically with preservation of normal tissues, with minimal on-target/off-tumor toxicity. CAR T cells as a monotherapy in clinical trials against various solid tumors have proven non-efficacious due in large part to immune escape (113). Combinatorial strategies with radiotherapy, chemotherapy, and other immunotherapies are promising to overcome the limitations of the immunosuppressive TME, while further investigation is warranted to optimize CAR T cell therapy for solid tumors.

## Author Contributions

Y-JL, LM, and ML wrote and revised the manuscript. Y-JL drew the figures. ML initiated the concept and supervised the writing. All authors contributed to the article and approved the submitted version.

## Conflict of Interest

ML has received research funding from Arbor, BMS, Accuray, Tocagen, Biohaven, Kyrin-Kyowa, Biohaven, Urogen. He also has been a consultant for Tocagen, VBI, InCephalo Therapeutics, Pyramid Bio, Merck, BMS, Insightec, Biohaven, Sanianoia, Hemispherian, Black Diamond Therapeutics, Novocure, Noxxon, and a shareholder of Egret Therapeutics.

The remaining authors declare that the research was conducted in the absence of any commercial or financial relationships that could be construed as a potential conflict of interest.

## Publisher’s Note

All claims expressed in this article are solely those of the authors and do not necessarily represent those of their affiliated organizations, or those of the publisher, the editors and the reviewers. Any product that may be evaluated in this article, or claim that may be made by its manufacturer, is not guaranteed or endorsed by the publisher.
